# Effects of *Scrophularia buergeriana* Extract (Brainon^®^) on Aging-Induced Memory Impairment in SAMP8 Mice

**DOI:** 10.3390/cimb45020084

**Published:** 2023-02-03

**Authors:** Hae Lim Kim, Sung Kwon Lee, Da Eun Min, Tonking Bastola, Bo Yoon Chang, Jin Hye Bae, Dong Ryung Lee

**Affiliations:** 1 Research Institute, NUON Co., Ltd., Jungwon-gu, Seongnam 13201, Republic of Korea; 2 Institute of Pharmaceutical Research and Development, College of Pharmacy, Wonkwang University, Iksan 54538, Republic of Korea

**Keywords:** *Scrophularia buergeriana*, Brainon^®^, cognitive dysfunction, SAMP8 mice, Aβ_1–42_/Tau hyperphosphorylation, antioxidant, anti-inflammation, anti-apoptotic, mito/autophagy, synaptic plasticity/neurotransmitter

## Abstract

Alzheimer’s disease (AD) is a worldwide problem. Currently, there are no effective drugs for AD treatment. *Scrophularia buergeriana* Miquel (SB) is a traditional herbal medicine used in Korea to treat various diseases. Our previous studies have shown that ethanol extract of SB roots (SBE, Brainon^®^) exhibits potent anti-amnesic effects in Aβ_1–42_- or scopolamine-treated memory impairment mice model and neuroprotective effects in a glutamate-induced SH-SY5Y cell model. In this study, we evaluated the therapeutic effects of Brainon^®^ and its mechanism of action in senescence-accelerated mouse prone 8 (SAMP8) mice. Brainon^®^ (30 or 100 mg/kg/day) was orally treated to six-month-old SAMP8 mice for 12 weeks. Results revealed that Brainon^®^ administration effectually ameliorated cognitive deficits in Y-maze and passive avoidance tests. Following the completion of behavioral testing, western blotting was performed using the cerebral cortex. Results revealed that Brainon^®^ suppressed Aβ_1–42_ accumulation, Tau hyperphosphorylation, oxidative stress, and inflammation and alleviated apoptosis in SAMP8 mice. Brainon^®^ also promoted synaptic function by downregulating the expression of AChE and upregulating the expression of p-CREB/CREB and BDNF. Furthermore, Brainon^®^ restored SAMP8-reduced expression of ChAT and -dephosphorylated of ERK and also decreased AChE expression in the hippocampus. Furthermore, Brainon^®^ alleviated AD progression by promoting mitophagy/autophagy to maintain normal cellular function as a novel finding of this study. Our data suggest that Brainon^®^ can remarkably improve cognitive deficiency with the potential to be utilized in functional food for improving brain health.

## 1. Introduction

Alzheimer’s disease (AD), a major form of dementia, is characterized by cognitive deficiency and progressive memory decline [1]. Worldwide, there are 47 million people currently suffer from dementia. These numbers are expected to rise to 131 million by 2050, raising the suffering of patients and the burden of health and social care systems [2].

AD has diverse pathological features. The most representative features are the accumulation of brain β-amyloid (Aβ) plaques outside neurons and hyperphosphorylation of tau protein known to cause nerve fiber masses such as tau tangles inside neurons, which are targets for drug development and diagnostic biomarkers [3]. Nerve fiber tangles and Aβ can influence microglial cells, cause oxidative stress and inflammatory responses, and cause abnormal glucose metabolism. Such oxidative stress, inflammation, and abnormal glucose metabolism can impair the function of mitochondria. In addition, the accumulation of damaged mitochondria can cause a chain of cascade reactions, eventually leading to neuronal death, which is one of the pathological causes of AD [4]. It has been reported that inhibition of mitophagy, an important form of macroautophagy, can increase neuronal damage. Therefore, mitophagy dysfunction is regarded as one of the major causes of neuronal damage in neurodegenerative illnesses such as AD [5,6]. In autophagy, the process of self-degradation within a cell, cytoplasmic contents such as damaged organelles and long-lived proteins are broken down by lysosomes [7]. Thus, autophagy plays an important role in the prevention of neurodegenerative illness. Moreover, autophagy dysfunction causes cellular aging and affects many other cells signaling [8]. In addition, depletion of the neurotransmitter acetylcholine (ACh) occurs in AD, and degeneration of cholinergic neurons is also an important factor contributing to the development of AD [9,10]. However, the mechanism of AD is extensive. It still remains elusive.

Senescence-accelerated mouse prone 8 (SAMP8) is a preferred animal model of aging for dementia and brain aging research. It has been proposed as a mammal model to study AD pathogenesis and to develop preventive and therapeutic strategies for age-related AD [11]. SAMP8 mice show AD features such as the deposition of Aβ and excessive Tau protein phosphorylation. They also show characteristics such as those of the elderly, such as shortened lifespan, hair loss, and decreased physical activity [12,13]. Therefore, SAMP8 mice and SAM resistant 1 (SAMR1) mice with the same genetic background have been utilized in AD studies. Recently, many studies have been conducted to delay aging using the SAMP8 model [14].

*Scrophularia buergeriana* Miquel (SB), a perennial herb belonging to the Scrophulariaceae family, is indigenous to Korea and found in China and Japan. In oriental traditional medicine, dried SB roots have long been utilized to treat many diseases such as high fever, swelling, constipation, pharyngitis, and neuro-inflammation [15]. SB roots contain various components, including 8-O-E-p-methoxycinnamoyl-harpagide (MCA-Hg), E-harpagoside, cinnamic acid, E-p-methoxy-cinnamic acid (p-MCA), and angoroside C [16,17,18,19]. In a previous study, we demonstrated the neuroprotective effects of an ethanol extract of SB roots (SBE; Brainon^®^) against glutamate-induced cytotoxicity on SH-SY5Y cells [17]. Furthermore, our previous study has shown that Brainon^®^ can exert anti-amnesic effects by inhibiting oxidative stress, apoptosis, and accumulation and hyperphosphorylation of Tau protein in memory-deficit mice induced by Aβ_1–42_ [18]. Moreover, in another study, we have proven that Brainon^®^ possesses antioxidant, anti-inflammatory, and anti-apoptotic effects through cAMP response element-binding (CREB) and brain-derived neurotrophic factor (BDNF) mechanism in a mice model induced by scopolamine [19]. However, the effect of Brainon^®^ in the SAMP8 mouse model has not been elucidated. Therefore, the purpose of this study was to investigate the effects of Brainon^®^ on aging-induced memory impairment and the mechanisms involved using SAMP8 and age-matched SAMR1 mice.

## 2. Materials and Methods

### 2.1. Chemicals and Reagents

Primary antibodies against β-secretase (BACE, Cat# 5606), phospho-Tau (p-Tau, Cat# 15013), Tau (Cat# 46687), Catalase (Cat# 12980), NOD-like receptor (NLR) family pyrin domain containing 3 (NLRP3, Cat# 13158), apoptosis-associated speck-like protein containing a caspase recruitment domain (ASC, Cat# 67824), Caspase-1 (Cat# 3866), Bcl-2-associated X protein (Bax, Cat# 2772), B-cell lymphoma 2 (Bcl-2, Cat# 3498), cleaved caspase-9 (Cat# 20750), cleaved caspase-3 (Cat# 9664), cleaved poly (ADP-ribose) polymerase (PARP, Cat# 5625), FUN14 Domain-Containing 1 (FUNDC1, Cat# 49240), PTEN-induced putative kinase 1 (PINK1, Cat# 6946), Parkin (Cat# 4211), Beclin1 (Cat# 3495), microtubule-associated protein 1A/1B-light chain 3B (LC3B, Cat# 3868), p-cAMP response element-binding (p-CREB, Cat# 9198), CREB (Cat# 9197), choline acetyltransferase (ChAT, Cat# 27269), p-extracellular signal-regulated kinase (p-ERK, Cat# 4377), ERK (Cat# 4695), and β-actin (Cat# 8457) were obtained from Cell Signaling Technology (Danvers, MA, USA). Superoxide dismutase-1 (SOD-1, Cat# sc-515404), SOD-2 (Cat# sc-137254), β-amyloid (Aβ_1–42_, Cat# sc-28365), glutathione peroxidase-1 (GPx-1, Cat# sc-133160), glutathione reductase (GR, Cat# sc-133245), and acetylcholinesterase (AChE, Cat# sc-373901) were purchased from Santa Cruz Biotechnology (Santa Cruz, CA, USA). Nitric oxide synthase (iNOS, Cat# PA1-036) was obtained from Invitrogen Life Technologies (Carlsbad, CA, USA). Tumor necrosis factor α (TNFα, Cat# ab66579), IL-6 (Cat# ab9324), interleukin-1β (IL-1β, Cat# ab9722), and anti-brain-derived neurotrophic factor (BDNF, Cat# ab108319) were purchased from Abcam Inc. (Cambridge, CA, USA). Horseradish peroxidase (HRP)-linked anti-rabbit IgG and HRP-linked anti-mouse IgG antibodies were purchased from GenDEPOT (Barker, TX, USA).

### 2.2. Sample Preparation

Brainon^®^ was supplied by NUON Co., Ltd., (Seongnam, Korea). SB roots were dried and then extracted utilizing 70% EtOH. The extracted solution was filtered, concentrated, and finally dried to yield SB extract (SBE; Brainon^®^). The detailed process was described in a previous report [17,18,19]. Brainon^®^ was dissolved in distilled water (DW) and then applied to in vivo studies.

### 2.3. HPLC Analysis

HPLC analysis of Brainon^®^ was carried out on a Shimadzu LC-40D system (Shimadzu, Kyoto, Japan). The HPLC mobile phase consisted of 1% acetic acid in water (solvent A) and acetonitrile (solvent B) at 0.7 mL/min flow through Waters Sunfire C18 column (4.6 × 250 mm, 5 μm). The column temperature was kept at 40 °C, the UV detection wavelength was 329 nm, and the injection amount was 10 μL.

### 2.4. Animals

Male senescence-accelerated mouse prone 8 (SAMP8) mice at 2 months old and their control strain SAM resistant 1 (SAMR1) mice were purchased from Central Lab Animal Inc. (Seoul, Korea). The animal room was maintained at a temperature of 23 ± 2 °C, an air ventilation of 10 to 20 times/h, a light intensity of 150 to 300 lux, and a 12 h–12 h of light–dark cycle. All studies were performed in accordance with guidelines for animal experimentation provided by Wonkwang University. They were approved by the Institutional Animal Care and Use Committee of Wonkwang University (No. WKU21-65).

### 2.5. Design of Experiment

Mice were utilized in the experiment once they reached 6 months old. Mice were randomly divided into five groups: SAMR1 group (R1), SAMP8 group (P8), SAMP8 + Brainon^®^ 30 mg/kg administered group (P8 + Brainon^®^ 30), SAMP8 + Brainon^®^ 100 mg/kg administered group (P8 + Brainon^®^ 100), and SAMP8 + *Ginkgo biloba* extract (GBE) 50 mg/kg administered group (P8 + GBE 50). GBE was used as a positive control agent because it could improve memory deficit. Brainon^®^ and GBE were dissolved in DW and administered (10 mL/kg/day) to mice for 12 weeks via gastric gavage. R1 and P8 groups were given the same volume of DW only.

### 2.6. Y-Maze Test

Spontaneous spatial recognition was evaluated in a Y-maze apparatus consisting of three identical arms (41 cm in length, 10 cm in width, and 25 cm in height). Each mouse was allowed to freely explore the three arms in the center of the maze for 8 min. The arm entries sequence was recorded by a camera right above the device. A spontaneous alternation was defined as arm choices different from the previous two choices (e.g., ABC, BCA, CAB, etc.). Alternation percentage (%) was calculated as a proportion of total spontaneous alternations to possible alternations (total arm entries − 2) × 100%.

### 2.7. Step-Through Passive Avoidance Task

The device (Gemini Avoidance System, San Diego Instruments, San Diego, CA, USA) used for the passive avoidance test consisted of two identical compartments separated into dark and light areas with an automated door and an electric floor. The experiment was conducted at the same time every day for two days. On the first day, mice were placed in a light compartment and acclimatized for 300 s. The door was automatically opened. Electrical stimulation of 0.4 mA for 3 s was applied when the mouse moved to a dark compartment. On the second day, the same experiment was repeated, measuring the time taken for the mice to move to a dark compartment, but electrical stimulation was not applied.

### 2.8. Extraction of Protein and Western Blot Analysis

The cerebral cortex and hippocampus were cut into small pieces. Proteins were extracted utilizing RIPA reagent (DYNE Bio, Seongnam, Korea). Supernatants were collected by centrifugation at 10,000× *g* at 4 °C for 15 min. Protein concentrations were determined utilizing the BCA protein assay kit (Thermo Fisher Scientific, Waltham, MA, USA). An equal amount of protein (30 µg) was subjected to sodium dodecyl-sulfate polyacrylamide gel electrophoresis (SDS-PAGE) and transferred onto polyvinylidene difluoride (PVDF) membranes (Millipore Corp., Bedford, MA, USA). Membranes were blocked with 5% skim milk at 23 °C for 1 h and then incubated with primary antibodies at 4 °C overnight. After washing with tris-buffered saline with tween-20 (TBS-T), membranes were then incubated with secondary antibodies at room temperature for 1 h. Membranes were then processed for detection utilizing enhanced chemiluminescence (ECL) solution (Atto, Tokyo, Japan). Band intensity was calculated utilizing the ImageJ program (National Institutes of Health, Bethesda, MD, USA).

### 2.9. Immunofluorescence Staining

The isolated hippocampi were post-fixed in 4% paraformaldehyde overnight, and subsequently immersed in 30% sucrose solution. After freezing, the brains were coronally sectioned into 35 μm hippocampal sections in a cryostat. Then, the sections were incubated with phosphate-buffered saline containing tween-20 (PBST) containing 4% bovine serum albumin (BSA) and 5% fetal bovine serum (FBS). After being reacted with primary ChAT antibody, the sections were exposed to Alexa Fluor 488-conjugated goat anti-rabbit IgH secondary antibody and stained utilizing DAPI. Sections were photographed with a confocal laser-scanning microscope (×20 magnification; Nikon, Tokyo, Japan). The density of ChAT staining was measured using the ImageJ program and statistically analyzed with GraphPad Prism (GraphPad Software, San Diego, CA, USA).

### 2.10. Statistical Analysis

Differences between the two groups were analyzed using Student’s *t*-test. A one-way analysis of variance followed by Tukey’s post hoc test was used for multiple comparisons. Results are expressed as mean ± standard deviation. Differences between groups were considered statistically significant at *p* < 0.05 and *p* < 0.01.

## 3. Results

### 3.1. Composition of Brainon^®^

**Figure 1:**
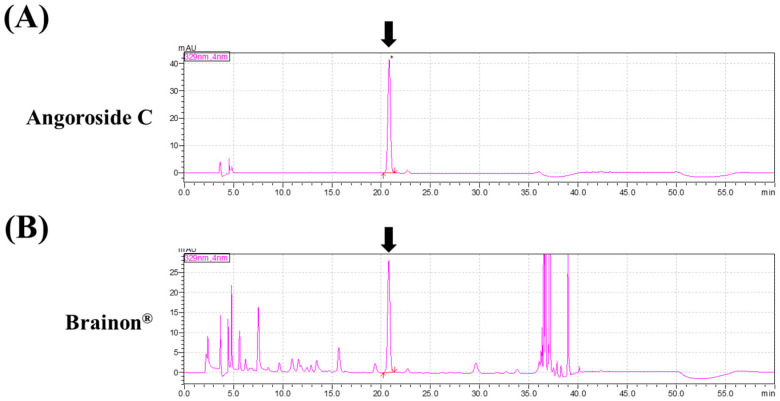
Composition of Brainon^®^. High-performance liquid chromatography (HPLC) chromatogram of Angoroside C and Brainon^®^. HPLC chromatogram of (**A**) Angoroside C (standard) and (**B**) Brainon^®^. The arrow indicates the peak for Angoroside C.

The marker compound within Brainon^®^ was identified by high-performance liquid chromatography (HPLC) analysis. As a result of quantitative analysis, the content of Angoroside C approximately 0.5% in Brainon^®^ was confirmed (Figure 1). Optimized Brainon^®^ was utilized for the following in vivo study.

### 3.2. Brainon^®^ Prevents Age-Dependent Cognitive Declines of SAMP8 Mice in the Y-Maze Test

**Figure 2:**
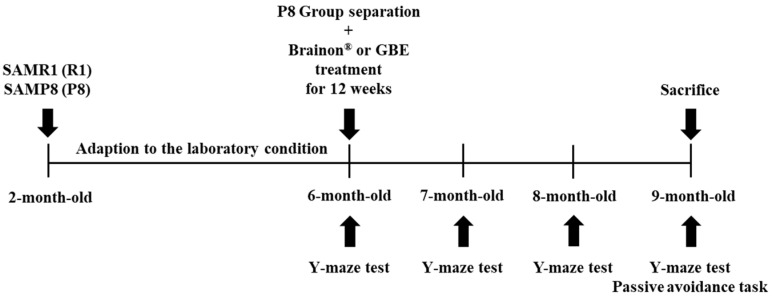
Schematic representation of the experimental design.

To evaluate the effects of Brainon^®^ on cognitive function, 6-month senescence-accelerated mouse prone 8 (SAMP8) mice and SAM resistant 1 (SAMR1) mice were administered with Brainon^®^ (30 or 100 mg/kg, p.o.), GBE (50 mg/kg, p.o.), or the same volumes of distilled water (DW), once daily for 12 weeks (Figure 2).

**Figure 3:**
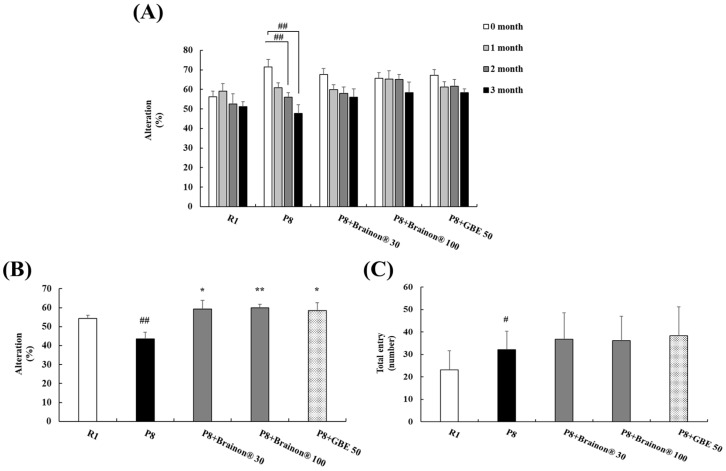
Brainon^®^ prevents age-dependent cognitive declines of SAMP8 mice in the Y-maze test. (**A**) SAMP8 considerably reduced the level of spontaneous alternation over the time period. (**B**) The average spontaneous alternation rate. (**C**) The number of total arm entries between SAMP8 group and Brainon^®^ group or GBE group were not statistically different. Data are expressed as mean ± SD of independent experiments (*n* = 6). # *p* < 0.05 and ## *p* < 0.01 vs. R1 (SAMR1) group; * *p* < 0.05 and ** *p* < 0.01 vs. P8 (SAMP8) group.

To analyze the gradual changes in memory function, the Y-maze test was carried out at 0, 1, 2, and 3 months after treatment initiation when the mice were 6, 7, 8, and 9 months old, respectively. Each mouse was allowed to freely explore the Y-maze for 8 min, the entries into each arm were scored, and the spontaneous alternation percentage was utilized to investigate spatial short-term working memory [20,21,22]. As shown in Figure 3A, the pattern of spontaneous alternation percentage in SAMP8 mice gradually decreased with increasing age. The alternation percentage was reduced significantly in 8- or 9-month-old SAMP8 mice in comparison with 6-month-old SAMP8 mice. However, there was no significant change within the other groups of normal SAMR1, Brainon^®^ 30 mg/kg, Brainon^®^ 100 mg/kg, or GBE 50 mg/kg over the time period from 6 months to 9 months. This showed that memory function decreased in the SAMP8 mice with increasing age while treatment with Brainon^®^ or GBE maintained the memory function over the time period. In the Y-maze test measured after 3 months of treatment in 9-month-old mice, the spontaneous alternation of untreated SAMP8 mice was lower in comparison with SAMR1 mice (Figure 3B). While SAMP8 mice treated with Brainon^®^ 30 mg/kg (*p* < 0.05) or Brainon^®^ 100 mg/kg (*p* < 0.01) showed considerably higher spontaneous alternation as compared with non-treated- or GBE-treated-SAMP8 mice. Interestingly, a significant difference was identified in the total number of arm entries in the SAMP8 group as compared with the SAMR1 group (Figure 3C), demonstrating that the general locomotor activity was higher in SAMP8 mice as comparison with SAMR1 [23,24]. However, there was no statistical significance between SAMP8 and Brainon^®^ or GBE groups. In addition, no correlation was observed between the Y-maze alternation and the total number of arm entries, implying that differences in the total number of arm entries did not affect the quantification of spontaneous alternation in the Y-maze. Overall, the Y-maze test showed that Brainon^®^ treatment inhibited memory deterioration over the time period in SAMP8 mice, and the effect was superior to GBE treatment.

### 3.3. Brainon^®^ Ameliorates Learning and Memory Impairments in the Passive Avoidance Task

**Figure 4:**
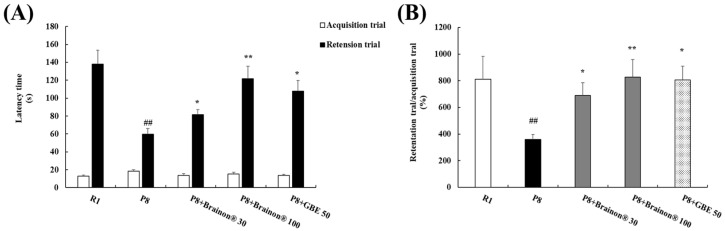
Brainon^®^ ameliorates learning and memory impairments in the passive avoidance task. (**A**) The latency times to enter the dark compartment were recorded in acquisition and retention trial. (**B**) The percentage ratio of the retention trial latency to acquisition trial latency was calculated for each mouse. Data are expressed as mean ± SD of independent experiments (*n* = 6). ## *p* < 0.01 vs. R1 (SAMR1) group; * *p* < 0.05 and ** *p* < 0.01 vs. P8 (SAMP8) group.

We assessed the effect of Brainon^®^ on learning and long-term memory using the step-through passive avoidance test in the SAMP8 mouse model (Figure 4). During the acquisition trial, each mouse placed in a bright compartment moved to a dark compartment habitually and received an electrical stimulation. A retention trial was performed 24 h after the acquisition trial and the latency time to re-enter the dark compartment was measured [25]. No significant differences were observed in latency time between groups during the acquisition trial (Figure 4A). In the retention trial, the SAMP8 group showed a considerably shorter latency time than the SAMR1 group, indicating memory impairments. The impairments were substantially reduced by administrated with Brainon^®^ 30 mg/kg (*p* < 0.05), Brainon^®^ 100 mg/kg (*p* < 0.01), or GBE 50 mg/kg (*p* < 0.05). Moreover, the percentage ratio of retention trial to acquisition trial, Brainon^®^ 30 mg/kg (*p* < 0.05), Brainon^®^ 100 mg/kg (*p* < 0.01), or GBE 50 mg/kg (*p* < 0.05) treatment considerably reversed the reduction of latency time (Figure 4B), suggesting the protective effect of Brainon^®^ from learning and long-term memory dysfunction in the SAMP8 aging model.

### 3.4. Effects of Brainon^®^ on Inhibition of Aβ_1–42_ Production and Hyperphosphorylation of Tau in the Cerebral Cortex of SAMP8 Mice

**Figure 5:**
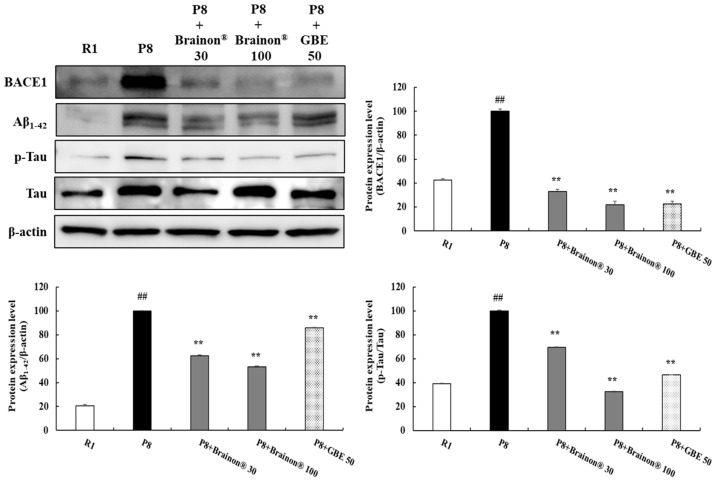
Effects of Brainon^®^ on inhibition of Aβ_1–42_ production and hyperphosphorylation of Tau in the cerebral cortex of SAMP8 mice. BACE1 (70 kDa), Aβ_1–42_ (4–46 kDa), p-Tau (50–80 kDa), and Tau (50–80 kDa) proteins were analyzed using Western blotting. The density of the protein band was measured using the ImageJ program. Protein was normalized with β-actin (45 kDa). Values are presented as mean ± SD (*n* = 3). ## *p* < 0.01 vs. R1 (SAMR1) group; ** *p* < 0.01 vs. P8 (SAMP8) group.

The main pathological markers in Alzheimer’s disease (AD) include β-amyloid (Aβ) accumulation and Tau hyperphosphorylation. β-secretase (BACE1) sequentially cleaves the amyloid A4 precursor protein (APP) to generate Aβ [26]. Therefore, the expression of BACE1, Aβ_1–42_, and p-Tau/Tau was assessed in this study. As shown in Figure 5, BACE1, Aβ_1–42_, and p-Tau/Tau levels were dramatically increased in the SAMP8 group (*p* < 0.01) compared with the SAMR1 group. However, treatment with Brainon^®^ 30 and 100 mg/kg (*p* < 0.01) greatly inhibited high levels of BACE1 expression in the SAMP8 group, suggesting that decreased accumulation of Aβ_1–42_ might be due to inhibitory effects of Brainon^®^ on BACE1. Moreover, compared with non-treated or GBE-treated SAMP8 mice, mice in Brainon^®^ 30 and 100 mg/kg treated groups exhibited significantly (*p* < 0.01) reduced Aβ_1–42_ expression. On the basis of the protective effect of Brainon^®^ on Aβ_1–42_ accumulation, we then determined the effect of Brainon^®^ on Tau phosphorylation in SAMP8 mice. Results on p-Tau/Tau indicated that treatment with Brainon^®^ 30 or 100 mg/kg (*p* < 0.01) significantly suppressed the phosphorylation of Tau in SAMP8 mice.

### 3.5. Brainon^®^ Promotes Antioxidant Enzymes in the Cerebral Cortex of SAMP8 Mice

**Figure 6:**
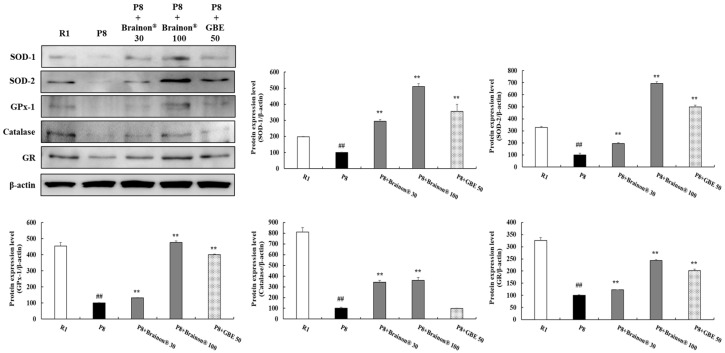
Brainon^®^ promotes antioxidant enzymes in the cerebral cortex of SAMP8 mice. SOD-1 (23 kDa), SOD-2 (25 kDa), GPx-1 (23 kDa), catalase (60 kDa), and GR (50–65 kDa) protein levels were analyzed utilizing Western blotting. Density of each protein band was measured using the ImageJ program. Protein was normalized with β-actin (45 kDa). Values are presented as mean ± SD (*n* = 3). ## *p* < 0.01 vs. R1 (SAMR1) group; ** *p* < 0.01 vs. P8 (SAMP8) group.

To assess the effects of Brainon^®^ on antioxidant enzymes in the cerebral cortex of SAMP8 mice, protein expression of superoxide dismutase-1 (SOD-1), SO D-2, glutathione peroxidase-1 (GPx-1), Catalase, and glutathione reductase (GR) were evaluated. Results revealed that protein expression of SOD-1, SOD-2, GPx-1, Catalase, and GR were markedly decreased in SAMP8 mice in comparison with SAMR1 mice. However, treatment with Brainon^®^ 30 or 100 mg/kg significantly (*p* < 0.01) increased levels of these antioxidant enzymes in SAMP8 mice, dose-dependently. Especially, Brainon^®^ 30 and 100 mg/kg treatment groups showed significantly (*p* < 0.01) higher expression levels of Catalase compared with non-treated or GBE treated-SAMP8 mice (Figure 6).

### 3.6. Brainon^®^ Inhibits Expression of Inflammatory Cytokines in the Cerebral Cortex of SAMP8 Mice

**Figure 7:**
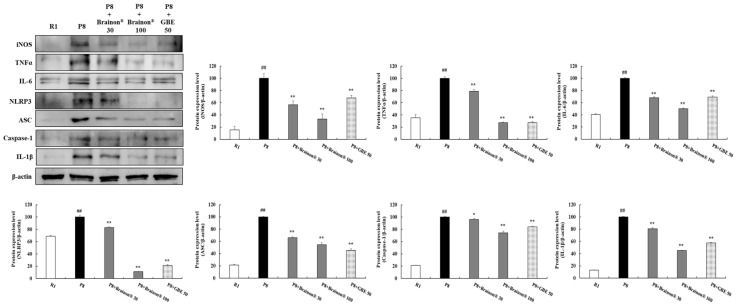
Brainon^®^ inhibits expression of inflammatory cytokines in the cerebral cortex of SAMP8 mice. iNOS (130 kDa), TNFα (25 kDa), IL-6 (17–25 kDa), NLRP3 (85–110 kDa), ASC (22 kDa), Caspase-1 (20–48 kDa), and IL-1β (30 kDa) protein levels were assessed using Western blotting. The density of each protein band was measured using the ImageJ program. Protein was normalized with β-actin (45 kDa). Values are presented as mean ± SD (*n* = 3). ## *p* < 0.01 vs. R1 (SAMR1) group; * *p* < 0.05 and ** *p* < 0.01 vs. P8 (SAMP8) group.

Given the relation of inflammation to AD, the effects of Brainon^®^ on the inflammatory status of aged SAMP8 brains were characterized. As shown in Figure 7, inflammatory status was increased in SAMP8 mice compared to SMAR mice. Protein expression of nitric oxide synthase (iNOS), tumor necrosis factor α (TNFα), and interleukin-6 (IL-6) were exceedingly (*p* < 0.01) enhanced in SAMP8 mice than in SAMR1 mice. However, administration of Brainon^®^ 30 mg/kg or 100 mg/kg (*p* < 0.01) greatly decreased protein expression of iNOS, TNFα, and IL-6 in SAMP8 mice. Notably, the expression levels of iNOS and IL-6 were significantly (*p* < 0.01) inhibited in Brainon^®^ 30 and 100 mg/kg treatment groups compared with non-treated or GBE-treated SAMP8 mice.

Furthermore, protein expression of NOD-like receptor (NLR) family pyrin domain containing 3 (NLRP3), apoptosis-associated speck-like protein containing a caspase recruitment domain (ASC), and Caspase-1 were considerably (*p* < 0.01) higher in SAMP8 mice comparison with SAMR1 mice. Interestingly, expression levels of IL-1β were also immensely higher in SAMP8 mice. However, treatment with Brainon^®^ significantly decreased expression levels of these proteins in SAMP8 mice (NLRP3, ASC, and IL-1β in Brainon^®^ 30 mg/kg or 100 mg/kg, *p* < 0.01; Caspase-1 in Brainon^®^ 30 mg/kg, *p* < 0.05; and Caspase-1 in Brainon^®^ 100 mg/kg, *p* < 0.01) (Figure 7).

### 3.7. Brainon^®^ Inhibits Apoptosis-Related Proteins in the Cerebral Cortex of SAMP8 Mice

**Figure 8:**
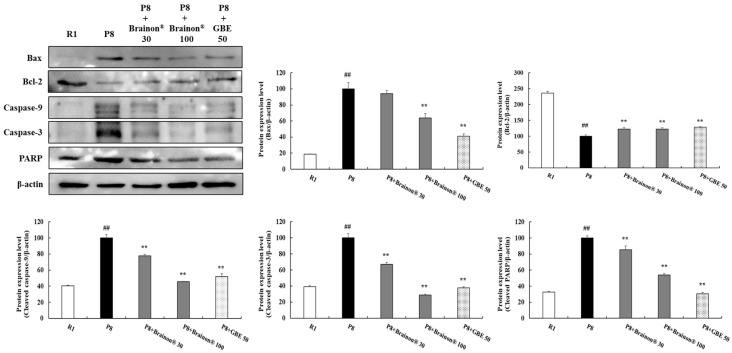
Brainon^®^ inhibits apoptosis-related proteins in the cerebral cortex of SAMP8 mice. Bax (20 kDa), Bcl-2 (26 kDa), cleaved caspase-9 (35 kDa), cleaved caspase-3 (17–19 kDa), and cleaved PARP (89 kDa) protein levels were analyzed using Western blotting. The density of each protein band was measured using the ImageJ program. Protein was normalized with β-actin (45 kDa). Values are presented as mean ± SD (*n* = 3). ## *p* < 0.01 vs. R1 (SAMR1) group; ** *p* < 0.01 vs. P8 (SAMP8) group.

To demonstrate the effects of Brainon^®^ on apoptosis, we examined expression levels of Bcl-2-associated X protein (Bax), B-cell lymphoma 2 (Bcl-2), cleaved caspase-9, cleaved caspase-3, and cleaved poly (ADP-ribose) polymerase (PARP) in the cerebral cortex of mice. As shown in Figure 8, protein expression of Bax, cleaved caspase-9, cleaved caspase-3, and cleaved PARP were immensely higher whereas the protein level of Bcl-2 was substantially lower in SAMP8 mice than in SAMR1 mice. However, treatment with Brainon^®^ 100 mg/kg (*p* < 0.01) or GBE 50 mg/kg (*p* < 0.01) greatly decreased the expression of Bax in SAMP8 mice. Furthermore, treatment with Brainon^®^ 30 mg/kg (*p* < 0.01), Brainon^®^ 100 mg/kg (*p* < 0.01), or GBE 50 mg/kg (*p* < 0.01) significantly decreased expression levels of cleaved caspase-9, cleaved caspase-3, and cleaved PARP but significantly increased Bcl-2 levels in SAMP8 mice.

### 3.8. Brainon^®^ Promotes Mitophagy/Autophagy in the Cerebral Cortex of SAMP8 Mice

**Figure 9:**
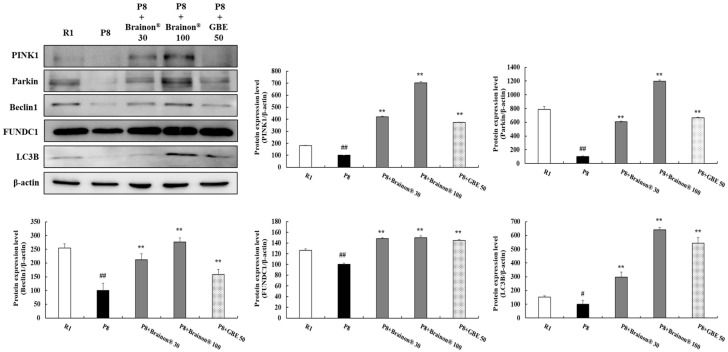
Brainon^®^ promotes mitophagy/autophagy in the cerebral cortex of SAMP8 mice. PINK1 (50–60 kDa), Parkin (50 kDa), Beclin1 (60 kDa), FUNDC1 (17 kDa), and LC3B (14–16 kDa) protein levels were analyzed using Western blotting. The density of each protein band was measured using the ImageJ program. Protein was normalized with β-actin (45 kDa). Values are presented as mean ± SD (*n* = 3). # *p* < 0.05 and ## *p* < 0.01 vs. R1 (SAMR1) group; ** *p* < 0.01 vs. P8 (SAMP8) group.

The PTEN-induced putative kinase 1 (PINK1)-Parkin signaling pathway is the most representative pathway mediating mitophagy [27,28,29,30]. Thus, protein expressions of PINK1 and Parkin were evaluated in the cerebral cortex. In our results, the protein levels of PINK1 and Parkin were substantially (*p* < 0.01) lower in SAMR8 mice than in SAMR1 mice. However, treatment with Brainon^®^ 30 mg/kg or 100 mg/kg (*p* < 0.01) considerably enhanced the protein expression of PINK1 and Parkin in SAMP8 mice (Figure 9). Expression levels of proteins related to autophagy including Beclin1, FUN14 Domain-Containing 1 (FUNDC1), and microtubule-associated protein 1A/1B-light chain 3B (LC3B) were then evaluated. As shown in Figure 9, Beclin1 (*p* < 0.01), FUNDC1 (*p* < 0.01), and LC3B (*p* < 0.05) protein expression levels were lower in SAMP8 mice compared to SAMR1 mice. However, treatment with Brainon^®^ 30 mg/kg or 100 mg/kg (*p* < 0.01) greatly increased protein expression levels of these autophagy marker proteins. Compared non-treated or GBE-treated SAMP8 group, expression levels of PINK1, Beclin1, and FUNDC1 were significantly (*p* < 0.01) increased in Brainon^®^ 30 and 100 mg/kg treatment groups.

### 3.9. Brainon^®^ Inhibits AChE Activity but Increases BDNF Expression and CREB Phosphorylation Levels in the Cerebral Cortex of SAMP8 Mice

**Figure 10:**
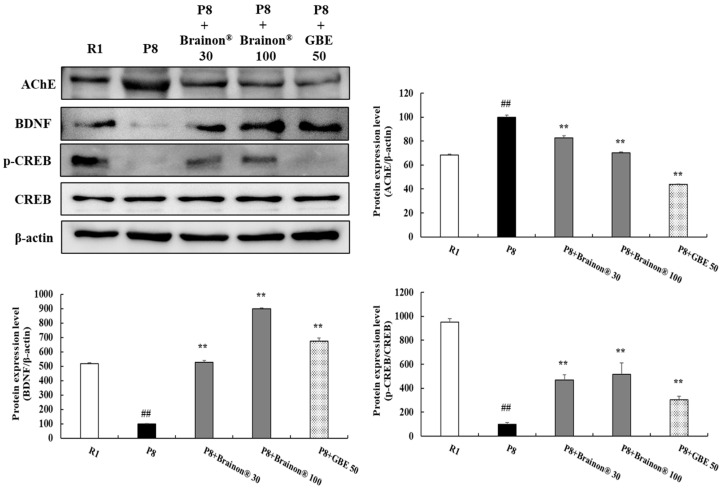
Brainon^®^ inhibits AChE activity but increases BDNF expression and CREB phosphorylation levels in the cerebral cortex of SAMP8 mice. AChE (68 kDa), BDNF (15 kDa), p-CREB (43 kDa), and CREB (43 kDa) protein levels were analyzed using Western blotting. The density of each protein band was measured using the ImageJ program. Protein was normalized with β-actin (45 kDa). Values are presented as mean ± SD (*n* = 3). ## *p* < 0.01 vs. R1 (SAMR1) group; ** *p* < 0.01 vs. P8 (SAMP8) group.

Results also presented that Brainon^®^ had a significant effect on synapse plasticity-related proteins in the cerebral cortex. Compared to the SAMP8 group, the SAMR1 group showed significantly (*p* < 0.01) higher expression levels of acetylcholinesterase (AChE). However, protein levels of AChE protein in the cerebral cortex of Brainon^®^ 30 mg/kg or 100 mg/kg (*p* < 0.01) treated SAMP8 mice were significantly decreased than in the cerebral cortex of SAMP8 mice without Brainon^®^ treatment. Moreover, to determine the effects of Brainon^®^ on the memory-associated proteins, we measured brain-derived neurotrophic factor (BDNF) expression and phospho-cAMP response element-binding (p-CREB)/CREB levels. As shown in Figure 10, protein expression of BDNF and p-CREB/CREB levels were significantly lower in SAMP8 mice (*p* < 0.01) than in SAMR1 mice. However, treatment with Brainon^®^ 30 or 100 mg/kg (*p* < 0.01) greatly increased protein expression of BDNF and p-CREB/CREB levels in SAMP8 mice, dose-dependently. Especially, mice with 30 and 100 mg/kg of Brainon^®^ treatment exhibited significantly (*p* < 0.01) upregulated p-CREB/CREB levels compared with non-treated or GBE-treated SAMP8 mice.

### 3.10. Brainon^®^ Regulates the Expressions of AChE and ChAT and the Phosphorylation of ERK in the Hippocampus of SAMP8 Mice

**Figure 11:**
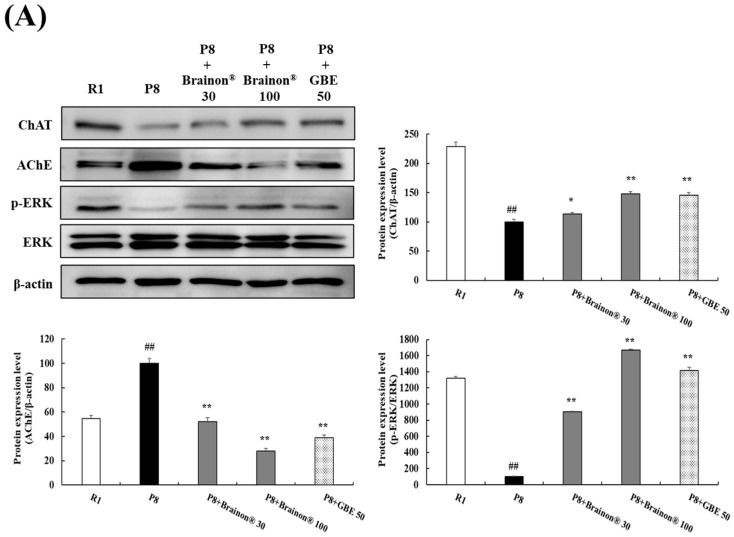

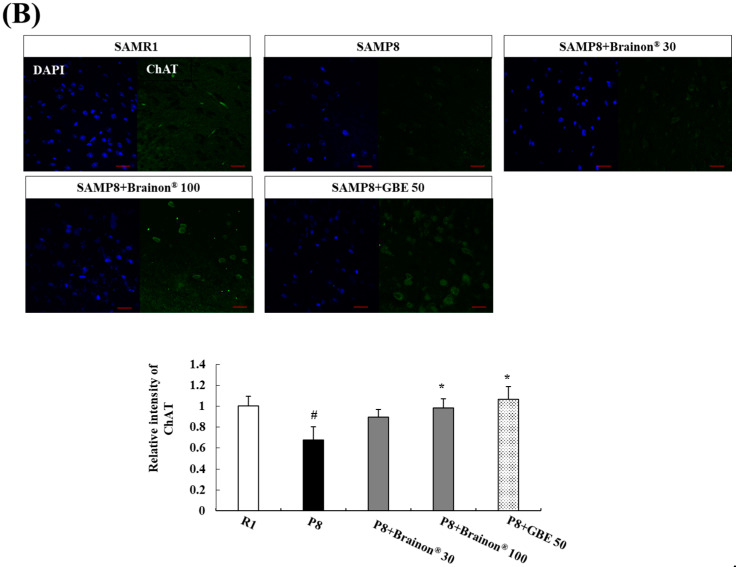
Brainon^®^ regulates the expressions of AChE and ChAT and the phosphorylation of ERK in the hippocampus of SAMP8 mice. (**A**) ChAT (71 kDa), AChE (68 kDa), p-ERK (42–44 kDa), and ERK (42–44 kDa) protein levels were analyzed using Western blotting. The density of each protein band was determined using the ImageJ program. Protein was normalized using β-actin (45 kDa). (**B**) Immunofluorescence staining of ChAT in the hippocampus of mice brain (×20 magnification). The density of ChAT staining was assessed with the ImageJ program and statistically analyzed by GraphPad Prism. Values are presented as mean ± SD (*n* = 3). # *p* < 0.05 and ## *p* < 0.01 vs. R1 (SAMR1) group; * *p* < 0.05 and ** *p* < 0.01 vs. P8 (SAMP8) group.

Recent studies suggested that active communication between the cortex and hippocampus converts new memories in the hippocampus into long-term memories stored in the cortex [31,32]. Choline acetyltransferase (ChAT) is one of the specific cholinergic marker proteins for the functional state of cholinergic neurons, which plays a crucial role to maintain the acetylcholine (ACh) levels in these neurons [33]. Therefore, ChAT and AChE are responsible for the synthesis and hydrolysis of acetylcholine, respectively. In this study, we further confirmed the effect of Brainon^®^ on ChAT and AChE expression in the hippocampus. Western blot analysis demonstrated that AChE expression, which was increased in the hippocampus of SAMP8 mice, was decreased by the co-treatment with Brainon^®^ 30 mg/kg (*p* < 0.05), 100 mg/kg (*p* < 0.01), or GBE 50 mg/kg (*p* < 0.01) (Figure 11A). On the other hand, the ChAT expression of the SAMP8 group (*p* < 0.01) decreased significantly in comparison with the SAMR1 group, whereas ChAT expression was upregulated by the co-treatment with Brainon^®^ 30 mg/kg (*p* < 0.05), 100 mg/kg (*p* < 0.01) or GBE 50 mg/kg (*p* < 0.01). Moreover, immunofluorescence staining of the hippocampus revealed that ChAT staining was significantly decreased in SAMP8 mice, which is upregulated by the co-treatment with Brainon^®^ 100 mg/kg or GBE 50 mg/kg (Figure 11B). These results suggest that Brainon^®^ inhibits AChE expression, increases ChAT expression, and thereby facilitates the cholinergic system, which might improve the aging-related cognitive deformity. In addition, the extracellular signal-regulated kinase (ERK) signaling pathway has been implicated in synaptic plasticity, and long-term memory formation. In addition, another study has shown that the phosphorylation of ERK is reduced in the aged SAMP8 mice compared with the SAMR1 group [34,35]. In the hippocampus of SAMP8 mice, ERK was considerably dephosphorylated compared with that of SAMR1 mice, and administration of Brainon^®^ 30 mg/kg, Brainon^®^ 100 mg/kg, or GBE 50 mg/kg reversed the aging-induced ERK dephosphorylation in the mouse hippocampus, suggesting the involvement of ERK signaling in Brainon^®^-induced alleviation against aging-related memory dysfunction.

## 4. Discussion

Alzheimer’s disease (AD) is characterized by progressive memory decline and declining cognitive impairment. Abnormal β-amyloid (Aβ) accumulation, neurofibrillary tangles associated with Tau protein hyperphosphorylation, oxidative injury, inflammation, apoptosis, dysfunction of autophagy and mitophagy, cholinergic neurons hypofunction, synapses loss, and loss of dendritic spines in the brain are features of pathological alterations of AD [5,6,8,36,37,38].

Previously, we have reported that *Scrophularia buergeriana* Miquel roots ethanol extract (SBE; Brainon^®^) exhibits potent anti-amnesic effects in Aβ_1–42_- or scopolamine-treated memory impairment mice model and neuroprotective effects in glutamate-induced SH-SY5Y cell model [17,18,19]. Senescence-accelerated mouse prone 8 (SAMP8) mouse model is a reliable animal model to study age-related AD pathogenesis and develop preventive and therapeutic strategies [11,39]. Hence, we administered Brainon^®^ to 6-month-old SAMP8 mice for 12 weeks. At 9 months of age, SAMP8 mice showed a stronger AD-related phenotype than SAM resistant 1 (SAMR1) mice. The preventive effect of Brainon^®^ on AD-related phenotype was prominent. Our results suggested that oral treatment of Brainon^®^ could considerably increase the learning ability and memory of SAMP8 mice.

Aβ is an important pathological feature in AD development. Accumulation of Aβ is the major cause of plaque formation. Aβ is produced from amyloid A4 precursor protein (APP) by sequential cleavage such as β-secretase (BACE1). Thus, downregulation of Aβ through inhibition of BACE1 can ameliorate cognitive deficits in SAMP8 mice by suppressing the formation of amyloid plaques [26]. In our study, BACE1 expression levels in the cerebral cortex of SAMP8 mice were substantially higher than in SMAR1 mice, consistent with the high expression of Aβ_1–42_ in the brains of SAMP8 mice. Brainon^®^ downregulated the protein levels of BACE1 which reduced expression levels of Aβ_1–42_. In particular, compared to SAMP8 mice treated with *Ginkgo Biloba* extract (GBE), the expression level of Aβ_1–42_ was significantly reduced in mice administered with both doses of Brainon^®^. Furthermore, there is growing evidence that Aβ, a major component of amyloid plaques, affects Tau phosphorylation [40]. Indeed, expression levels of p-Tau/Tau in the cerebral cortex of SAMP8 mice were suppressed by the administration of Brainon^®^ compared to those in SAMP8 mice without Brainon^®^ treatment.

Accumulation of Aβ can also promote oxidative stress in the intermembrane and cause reactive oxygen species (ROS) production by serving as a source of ROS [41,42,43]. The antioxidant defense system uses various enzymes such as superoxide dismutase (SOD), glutathione peroxidases (GPx), catalase, and glutathione reductases (GR) to protect against ROS attacks [44,45,46,47]. SOD is known as the first detoxification enzyme and the most powerful endogenous antioxidant. This enzyme acts as a catalyst to convert superoxide (O_2_^−^) radicals into ordinary oxygen (O_2_) and hydrogen peroxide (H_2_O_2_), making deleterious superoxide anion less harmless. GPx is also an important intracellular enzyme that can break down H_2_O_2_ to H_2_O with glutathione (GSH) as a substrate and convert GSH to glutathione disulfide (GSSG) through oxidation [48]. Catalase is also a common enzyme that catalyzes the breakdown of H_2_O_2_ into H_2_O and O_2_, consequently completing the detoxification process imitated by SOD [49,50]. GR is known to maintain reduced GSH levels in the redox cycle. This enzyme resists oxidative stress and maintains a reducing environment inside the cell [48]. Therefore, antioxidant defense systems play a crucial role in preventing ROS generation and regulating steady-state O_2_ concentration. Our results demonstrated that protein levels of SOD-1, SOD-2, GPx-1, Catalase, and GR were lower in SAMP8 mice in comparison with SAMR1 mice. However, Brainon^®^ treatment significantly increased expression levels of these antioxidant enzymes in SAMP8 mice. Notably, the increase in Catalase expression level was superior in mice administered with both doses of Brainon^®^ compared to GBE-treated SAMP8 mice.

There is increasing evidence showing that immoderate oxidative stress can immediately destroy cell membranes, protein, and RNA and elevate neuroinflammation and neuronal apoptosis [51]. Inflammatory responses include the inducement of inducible nitric oxide synthase (iNOS) and pro-inflammatory cytokines such as tumor necrosis factor α (TNFα), and interleukin-6 (IL-6) [52]. In the present study, the elevation of the inflammatory status was observed in SAMP8 mice based on protein expression of iNOS, TNFα, and IL-6. However, the increased inflammatory status in SAMP8 mice was decreased by treatment with Brainon^®^. The expression of iNOS and IL-6 were significantly suppressed in both dose groups of Brainon^®^ compared to GBE-treated SAMP8 mice. IL-1β, another pro-inflammatory cytokine that plays a crucial role in inflammatory responses of AD, has been reported to be regulated by NOD-like receptor (NLR) family pyrin domain containing 3 (NLRP3) inflammasome [53]. As an important class of cellular adapter protein, apoptosis-associated speck-like protein containing a caspase recruitment domain (ASC) is associated with the organization of the NLRP3 inflammasome. ASC has two domain structures: an effect domain (CARD) and an oligomeric domain (PYD). Through these two domains, ASC can recruit Caspase-1 precursors and NLRP3 to develop the NLRP3 inflammasome [54]. Caspase-1 can initiate and carry out a series of cellular processes to induce inflammatory reactions and apoptosis. After NLRP3 inflammasome assembly, the Caspase-1 precursor is utilized to catalyze the generation of active Caspase-1. Caspase-1 is an IL-1β converting enzyme, an effector of inflammatory cells that can mediate the conversion of inactive IL-1β precursor to mature IL-1β [55,56,57]. In this study, expression levels of NLRP3, ASC, Caspase-1, and IL-1β proteins were substantially higher in SAMP8 mice than in SAMR1 mice. However, treatment Brainon^®^ significantly decreased expression levels of these proteins in SAMP8 mice.

Increased oxidative stress and inflammatory response that occurs during AD might subsequently lead to neuronal death by apoptotic pathways [58]. Mitochondria are central to the regulation of apoptosis. Pro-apoptotic protein Bcl-2-associated X protein (Bax) can translocate to mitochondria and interact with the B-cell lymphoma 2 (Bcl-2) family to control the process of apoptosis. Bax can activate down-stream effector caspases such as cleaved caspase-9, cleaved caspase-3, and cleaved poly (ADP-ribose) polymerase (PARP). In contrast, the anti-apoptotic protein Bcl-2 can suppress the release of cytochrome C from mitochondria [59,60]. In our study, expression levels of apoptosis-related proteins such as Bax, cleaved caspase-9, cleaved caspase-3, and cleaved PARP were increased whereas protein expression of Bcl-2 was more suppressed in SAMP8 mice than in SMAR1 mice. However, treatment with Brainon^®^ increased Bcl-2 expression while decreasing the expression of apoptosis-related proteins in SAMP8 mice.

PTEN-induced putative kinase 1 (PINK1) is a serine/threonine-protein kinase capable of inducing mitophagy to remove damaged mitochondria. Parkin, a cytoplasmic E3 ligase, is crucial for maintaining mitochondrial morphology. Therefore, PINK1 and Parkin were the critical proteins of mitophagy to remove excess and impaired mitochondria [27]. Recent reports have also suggested that PINK1 and Parkin are reduced in AD progress [28]. Our results revealed that Brainon^®^ could alleviate the reduction of PINK1 and Parkin expression in the cerebral cortex of SAMP8 mice. Autophagy is a multi-step process that involves initiation, membrane nucleation, formation of the phagophore, expansion of the phagophore, fusion with the lysosome, and degradation. Beclin1 is a core component of the class III PI3K complex that regulates the maturation of autophagosomes and promotes autophagy. FUN14 domain-containing 1 (FUNDC1), a novel type of mitochondrial membrane protein, is located in the outer membrane of mitochondria that can mediate mitochondrial autophagy by interacting with LC3B, a key molecule in autophagy [29,30]. Our studies indicated that protein expression of autophagy-related proteins containing Beclin1, FUNDC1, and LC3B were decreased in SAMP8 mice than in SMAR1 mice. However, treatment with Brainon^®^ upregulated protein expression of Beclin1, FUNDC1, and LC3B in SAMP8 mice. Compared to GBE-treated SAMP8 mice, both dose groups of Brainon^®^ showed excellent upregulation of PINK1, Beclin1, and FUNDC1 expression.

Acetylcholine (ACh) which plays a crucial role in the central nervous system, is able to establish synaptic connections between neurons. It is involved in memory and cognition [61]. ACh is hydrolyzed by acetylcholinesterase (AChE). When AChE is inhibited, ACh levels will increase, which can ameliorate cholinergic deficiency, leading to improved memory and learning [10]. Brain-derived neurotrophic factor (BDNF), the most abundant neurotrophic factor in the brain, is broadly expressed in neurons. It is important for long-term memory because it can promote synaptic plasticity and neuronal survival. BDNF is regulated by cAMP response element-binding (CREB), which depends on synaptic activity. When synaptic activity increases, more BDNF is synthesized [62,63,64]. In the present study, increased protein expression of AChE and lower expression levels of BDNF and p-CREB/CREB levels were found in SAMP8 mice than in SAMR1 mice, which might have resulted from the impaired synaptic transmission. However, Brainon^®^ treatment significantly decreased AChE levels and increased expression levels of p-CREB/CREB and BDNF in SAMP8 mice. Notably, the increase in p-CREB/CREB levels was superior in both dose groups of Brainon^®^ compared with GBE-treated SAMP8 mice. Therefore, amelioration of memory and learning in SAMP8 mice were accompanied by increases in synapse plasticity-related protein BDNF and phosphorylation of CREB with a decrease in AChE in the brain.

In addition, with the cerebral cortex, the hippocampus also plays a crucial role in memory. Especially, the hippocampus seems to play an essential role in the formation of memory networks in the association cortex [65]. Therefore, in this study, the effect of Brainon^®^ on the hippocampal tissue was further examined. Choline acetyltransferase (ChAT) is one of the specific cholinergic marker proteins for the functional status of cholinergic neurons, which play an essential role to maintain acetylcholine (ACh) levels in neurons [33]. In the present study, ChAT levels and phospho-extracellular signal-regulated kinase (p-ERK) were decreased, whereas increased levels of AChE were found in SAMP8 mice in comparison with SAMR1 mice. However, Brainon^®^ treatment significantly increased expression levels of ChAT and p-ERK/ERK and decreased AChE levels in SAMP8 mice. These results also reveal that Brainon^®^ can ameliorate age-related cognitive malformations by suppressing AChE expression and increasing ChAT and p-ERK expression to promote the cholinergic system.

**Figure 12:**
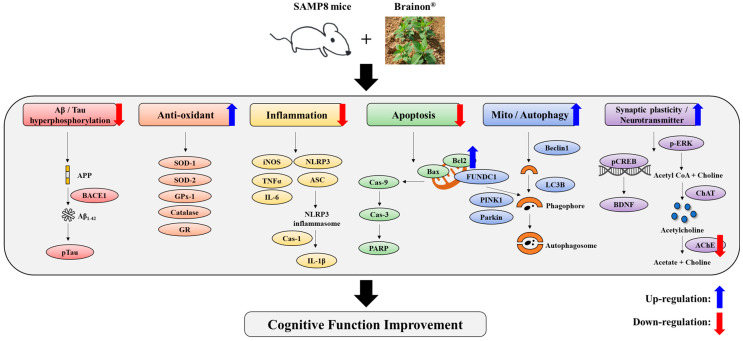
Schematic representation of obtained results and protective effects of Brainon^®^ to improve cognitive function in the SAMP8 mouse model.

In the present study, the action mechanism of Brainon^®^ was assessed regarding Aβ_1–42_ accumulation, Tau hyperphosphorylation, oxidative stress, anti-inflammation, neuronal cell death, mitophagy, autophagy, and synaptic plasticity. A detailed description is shown in Figure 12.

## 5. Conclusions

In summary, the present study demonstrates that dietary supplementation of Brainon^®^ can ameliorate cognitive deficiency and memory loss in SAMP8 mice. The mechanism identified here suggests that Brainon^®^ can depress accumulation of Aβ_1–42_, Tau hyperphosphorylation, oxidative stress, and inflammation and suppress apoptosis in the cerebral cortex. Brainon^®^ can also promote synaptic function by upregulating levels of p-CREB/CREB and BDNF. Especially, the novel finding of this study is that Brainon^®^ can alleviate AD progression by promoting mitophagy/autophagy to maintain normal cellular function. In addition, 30 and 100 mg/kg doses of Brainon^®^ treatments showed remarkable improvement in Aβ_1–42_, Catalase, iNOS, IL-6, PINK1, Beclin1, FUNDC1 expression, and p-CREB/CREB levels compared to the positive control GBE. These results also support the protective effect of low-dose Brainon^®^ against AD. Moreover, Brainon^®^ restored SAM8-reduced expression of ChAT and -decreased expression of AChE and -dephosphorylated of ERK in the hippocampus. Accordingly, Brainon^®^ can considerably improve cognitive deficiency with the potential to be utilized in functional foods for improving brain health.

## Data Availability

Not applicable.
